# Nanocrystalline Transition-Metal Gallium Oxide Spinels from Acetylacetonate Precursors via Solvothermal Synthesis

**DOI:** 10.3390/ma12050838

**Published:** 2019-03-12

**Authors:** Daniel S. Cook, Reza J. Kashtiban, Klaus Krambrock, Geraldo M. de Lima, Humberto O. Stumpf, Luciano R. S. Lara, José D. Ardisson, Richard I. Walton

**Affiliations:** 1 Department of Chemistry, University of Warwick, Coventry CV4 7AL, UK; danielseancook@gmail.com; 2 Department of Physics, University of Warwick, Coventry CV4 7AL, UK; R.Jalilikashtiban@warwick.ac.uk; 3 Departamento de Física, Universidade Federal de Minas Gerais, UFMG, Avenida Antônio Carlos 6627, Belo Horizonte MG, CEP 31270-901, Brazil; klaus@fisica.ufmg.br; 4 Departamento de Química, Universidade Federal de Minas Gerais, UFMG, Avenida Antônio Carlos 6627, Belo Horizonte MG, CEP 31270-901, Brazil; delima.geraldo@gmail.com (G.M.d.L.); stumpfho@gmail.com (H.O.S.); larovzki@gmail.com (L.R.S.L.); 5 Centro de Desenvolvimento em Tecnologia Nuclear, CDTN/CNEN, Avenida Antônio Carlos 6627, Belo Horizonte MG, CEP 31270-901, Brazil; jdr@cdtn.br

**Keywords:** ferrite, crystallisation, magnetism, XANES, Mössbauer spectroscopy, EPR

## Abstract

The synthesis of mixed-metal spinels based on substituted γ-Ga_2_O_3_ is reported using metal acetylacetonate precursors in solvothermal reactions with alcohols as solvents at 240 °C. New oxides of Cr, Mn and Fe have been produced, all of which are formed as nanocrystalline powders, as seen by high-resolution transmission electron microscopy (HR-TEM). The first chromium-gallium mixed oxide is thus formed, with composition 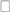
_0.33_Ga_1.87_Cr_0.8_O_4_ (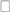 = vacant site). X-ray absorption near-edge spectroscopy (XANES) at the chromium K-edge shows the presence of solely octahedral Cr^3+^, which in turn implies a mixture of tetrahedral and octahedral Ga^3+^, and the material is stable on annealing to at least 850 °C. An analogous manganese material with average chemical composition close to MnGa_2_O_4_ is shown to contain octahedral Mn^2+^, along with some Mn^3+^, but a different inversion factor to materials reported by conventional solid-state synthesis in the literature, which are known to have a significant proportion of tetrahedral Mn^2+^. In the case of iron, higher amounts of the transition metal can be included to give an Fe:Ga ratio of 1:1. Elemental mapping using energy dispersive X-ray spectroscopy on the TEM, however, reveals inhomogeneity in the distribution of the two metals. This is consistent with variable temperature ^57^Fe Mössbauer spectroscopy that shows the presence of Fe^2+^ and Fe^3+^ in more than one phase in the sample. Variable temperature magnetisation and electron paramagnetic resonance (EPR) indicate the presence of superparamagnetism at room temperature in the iron-gallium oxides.

## 1. Introduction

The AB_2_X_4_ spinel structure, containing mixtures of cations A and B distributed over octahedral and tetrahedral sites in a (idealised) cubic-closed array of anions X, is well documented in the scientific literature, from its occurrence in various minerals, through the wide range of chemical compositions possible, to physical properties that find use in a range of practical applications [1]. The apparently simple empirical formula can accommodate various structural permutations: including degrees of inversion of the A and B cations over the tetrahedral and octahedral sites, the presence of cation deficiency, such as in M_2_O_3_ sesquioxides (= 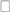
_0.33_M_2.67_O_4_ with vacant sites 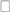) and migration of the cations to usually unoccupied sites within the close-packed anion array. These possibilities mean that structural disorder may be present that requires careful structural analysis using probes of both long-range and, especially, local-range order. Transition-metal cations are commonly found in oxide spinels, with their specific preferences for octahedral and tetrahedral coordination leading to physical properties that can potentially be tuned by composition. This can lead to, for example, cooperative magnetism, electronic and catalytic properties. The case of ferrites particularly exemplifies the interest, and applications, in magnetism of spinel oxides [2]. Contemporary applications in the field of energy are heavily researched in spinel-structured materials, such as in photocatalysis [3], electrocatalysis [4] and battery cathode materials [5].

Solution-mediated synthesis routes to oxide materials are particularly versatile, especially when direct crystallisation from the solution can be engineered without a need for annealing to bring about long-range order [6,7,8,9,10]. This may allow access to compositions that are not accessible using the high temperatures associated with classical solid-state chemistry and to allow crystal morphology (size and shape) to be dictated by the use of synthetic condition. Of particular focus is the formation of nanocrystalline materials; these may have a high surface: bulk structure and can show unusual electronic and magnetic effects unique to their constrained size. Solvothermal synthesis, borrowing from the solution chemistry used to prepare open-framework zeolite materials, has thus been explored has a versatile method for crystallisation of dense oxide (and chalcogenide) materials [6,7,8,9,10]. There are many variables in this approach to materials formation, which can collectively provide subtle control over the outcome of reaction, from the choice of solvent, the specific reagents used, the use of solution additives (for example, pH modifiers, surfactants, capping agents, sacrificial templating species), the source of energy (conventional heating, microwaves, ultrasound or electrochemical) as well as temperature (and gradients of temperature), pressure and simply the duration of reaction. The complex interplay of these synthetic variables remains poorly understood with the outcome of new reactions difficult to predict. This means that further exploration of solvothermal crystallisations of oxides are merited, with the potential for developing unconventional routes for the discovery of new and useful materials. 

Ga_2_O_3_ is an apparently simple oxide that shows complex polymorphism [11], whose electronic and catalytic properties have been the focus of growing attention in the past few years [12]. Among the various polymorphs is γ-Ga_2_O_3_, an oxide-deficient spinel structure with Ga^3+^ distributed among the tetrahedral and octahedral sites, the proportion of which depends upon the particle size of the material, especially when very small particles may have a surface rich in octahedral sites [13]. The material is conveniently produced using solvothermal methods directly from gallium metal [14], to which other transition-metal cations can be added as salts dissolved in the precursor solution to form mixed-metal gallium oxides [15,16,17]. Herein we report a new solvothermal route to transition-metal containing gallium oxides by using acetylacetonate precursors and organic solvents. This has allowed the preparation of new examples of Mn- and Fe-containing gallium oxide spinels, and the first chromium-gallium oxide, and we investigate the oxidation state distributions and magnetism of the materials using a variety of methods.

## 2. Experimental Section

### 2.1. Materials Synthesis

For the synthesis of Cr-substituted Ga_2_O_3_, 0.3 g (0.82 mmol) of Ga(acac)_3_ (Sigma-Aldrich, 99.99%, Gillingham, UK) and 0.093 g (0.35 mmol) of CrCl_3_·6H_2_O (Sigma-Aldrich, 96%, Gillingham, UK) were added into a 23 mL PTFE-lined steel autoclave followed by of 10 ml 1,4-butanediol (Alfa Aesar, 99%, Heysham, UK). If larger amounts of Cr were used, then there was always the formation of CrOOH present in the isolated polycrystalline material. The mixture was stirred vigorously for 15 min at room temperature before being sealed and placed into a fan-assisted oven pre-heated to 240 °C for 72 h. The autoclave was then removed from the oven and left to cool naturally to ambient temperature. The crystallised material was stirred in its mother liquor, aided by the addition of acetone to reduce the viscosity of the solvent. The material was collected by vacuum filtration and washed with copious amounts of acetone to yield a green coloured solid. The material was then dried overnight at 70 °C prior to characterisation.

For the preparation of Mn-substituted Ga_2_O_3_, Mn(acac)_3_ was used as a precursor. This was prepared using a method based on that described by Bhattacharjee et al. [18]. Five grams (31.7 mmol) of KMnO_4_ (Fisher Scientific, 99%, Loughborough, UK) were dissolved in the minimum amount of water aided by warming in a steam bath. Then, 22.0 g (220.0 mmol) of acetylacetone (Fisher Scientific, 99%, Loughborough, UK) was then added to the solution with vigorous stirring. The mixture was stirred for 20 min under reflux and then cooled for 30 min to effect the precipitation of dark brown crystals of Mn(acac)_3_. These crystals were filtered by vacuum filtration and then washed several times with an acetone:water mixture (1:1 by volume). The crystals were then recrystallised by dissolving them in the minimum amount of hot toluene followed by addition of petroleum ether and cooled to around 0 °C. For the oxide synthesis, 0.4 g (1.09 mmol) of Ga(acac)_3_ (Sigma-Aldrich, 99.99%, Gillingham, UK) and 0.165 g (0.468 mmol) of Mn(acac)_3_ were placed in a 23 mL PTFE liner with the subsequent addition of 10 mL 2-propanol. A higher Mn:Ga ratio in synthesis gave impurities in the product. The mixture was stirred at room temperature for 10 min before being sealed in a steel autoclave and placed in a fan-assisted oven pre-heated to 240 °C for 24 h. After the reaction time, the autoclave was removed from the oven and allowed to cool naturally to ambient temperature and the solid product collected by vacuum filtration. This was then washed with copious amounts of acetone to yield a light brown powder. The isolated solid polycrystalline material was then dried overnight at 70 °C prior to characterisation. 

Fe-substituted Ga_2_O_3_ was prepared in a similar method but using Fe(acac)_3_ (Sigma-Aldrich, 99.99%, Gillingham, UK) and Ga(acac)_3_ with 1,4-butanediol as solvent and 48 h of reaction time at 240 °C. The solids were recovered and dried in the same way as above. Spinel-structured materials with up to a 1:1 ratio of Fe:Ga could be produced in the reactions, and two samples were prepared for comparison, with ratios of Fe:Ga of 3:7 and 1:1.

### 2.2. Materials Characterisation

Powder XRD patterns were recorded at room temperature using a Panalytical X’Pert Pro MPD diffractometer (Malvern Panalytical, Malvern, UK) operating with monochromatic Cu Kα1 radiation and equipped with a PIXcel solid-state detector (Malvern Panalytical, Malvern, UK). Full pattern analysis of powder patterns was performed using the Pawley method within TOPAS software (TOPAS-Academic V6) to determine lattice parameters [19].

Scanning transmission electron microscopy (STEM) was performed using a JEOL ARM200F double aberration corrected instrument (Welwyn Garden City, UK) operating at 200 kV. Specimens were dispersed by ultrasound in ethanol and dropped onto 3 mm lacey carbon grids supplied by Agar Scientific (Stansted, UK) Annular dark field STEM (ADF-STEM) images were obtained using a JEOL annular field detector at a probe current of ∼23 pA with a convergence semi-angle of ∼25 mrad. Energy dispersive x-ray spectroscopy (EDS) measurements were carried out with an Oxford Instruments X-Max^N^ 100TLE windowless silicon drift detector (Abingdon, UK) to determine the elemental composition and distribution. 

A superconducting quantum interference device (SQUID) was used to measure the magnetic susceptibly of the Cr- and Mn-containing samples. Two instruments were used: a Quantum Design MPMS XL7 SQUID (Leatherhead, UK) and a Quantum Design MPMS-5S SQUID (Leatherhead, UK). Samples (5–25 mg) were placed inside a small gel capsule, in turn placed inside a plastic straw. Experimental measurement was carried out between 5 and 300 K with an applied magnetic field of 1000 Oe. For the iron-gallium oxide, static magnetic measurements as a function of temperature were performed in a commercial SQUID magnetometer. Zero-field-cooled (ZFC) and field-cooled (FC) curves were taken between 4.2 and 300 K, for a cooling field *H*_FC_ of 100 Oe. Data were obtained by first cooling the sample from room temperature in zero applied field (ZFC process) to the lowest temperature (4.2 K). Then, a field of 100 Oe was applied and the variation of magnetisation was measured with increasing temperature up to 300 K. After the last point was measured, the sample was cooled again to the lowest temperature keeping the same field (FC process); then, the *M* *vs T* data were measured for increasing temperatures. Hysteresis curves were obtained varying the magnetic field at fixed temperatures.

Electron paramagnetic resonance (EPR) experiments were performed on a commercial (MiniScope 400, Magnettech, Berlin, Germany) X-band (9.44 GHz) EPR spectrometer coupled to helium flux cryosystem (ESR 900, Oxford Instruments, Abingdon, UK) permitting sample temperatures to be varied between 4.2 and 350 K. Powdered samples were measured inside borosilicate tubes (Wilmad-LabGlass, Vineland, USA).

Mössbauer spectroscopy measurements were performed with a constant-acceleration spectrometer in transmission geometry, using a ^57^Co source in a Rh matrix (RITVERC Isotope Products, Starnberg, Germany) with activity ~50 mCi between 30 and 300 K. Hyperfine parameters such as the distribution of hyperfine magnetic field, isomer shift and quadrupole shift were determined by the NORMOS program, and α-Fe at 300 K was used to calibrate isomer shifts and velocity scale.

X-ray absorption near-edge spectroscopy (XANES) spectra at the Cr and Mn K-edges were recorded using Beamline B18 of the Diamond Light Source, Harwell, UK [20]. Data were collected from samples diluted with appropriate amounts of polyethylene powder and pressed in self-supporting discs around 1 mm thick in transmission mode. Incident energies were selected using a water-cooled, fixed-exit, double-crystal monochromator with Si(111) crystals. The beam was focused horizontally and vertically using a double toroidal mirror, coated with Pt, 25 m from the source, while a pair of smaller plane mirrors were used for harmonic rejection. The raw data were normalised using the software ATHENA (version 0.9.26) [21] to produce XANES spectra.

## 3. Results and Discussion

### 3.1. Cr-Ga_2_O_3_

**Figure 1:**
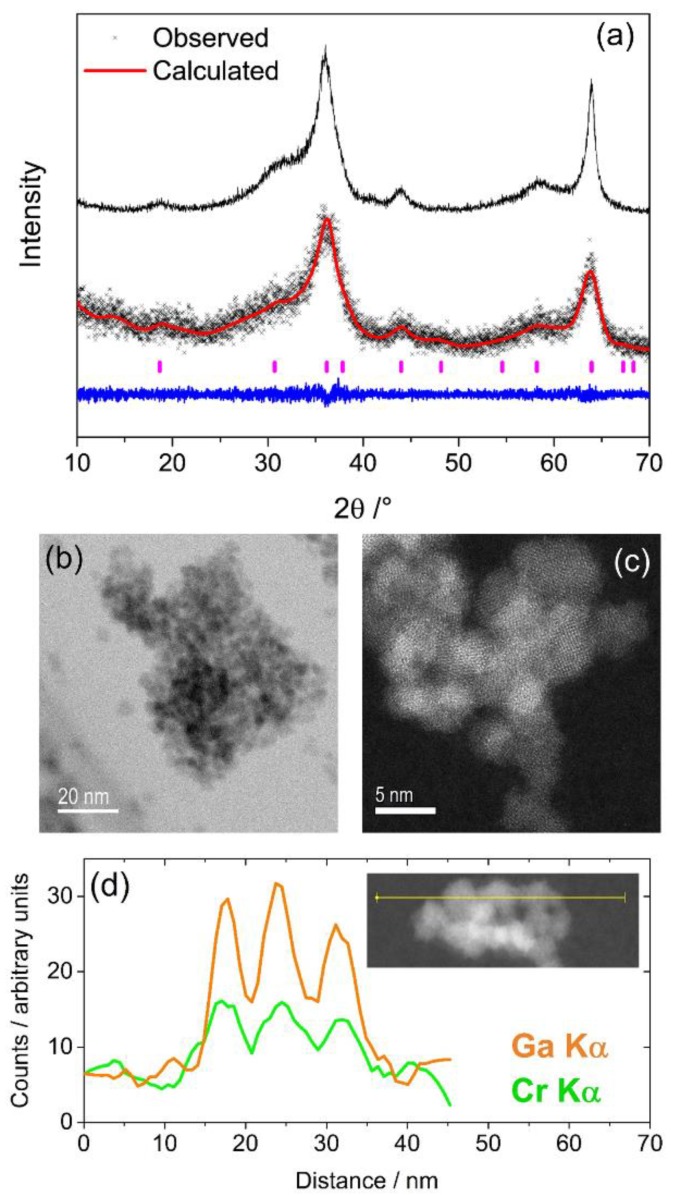
(**a**) Pawley refinement of the X-ray diffraction profile (*λ* = 1.5406 Å) of the chromium substituted γ-Ga_2_O_3_ with the pattern of pure γ-Ga_2_O_3_ above as a comparison. The red line is the fitted profile, the black points the measured data and the blue ticks the positions of allowed Bragg peaks. The blue line is the difference curve. (**b**,**c**) High-resolution transmission electron microscopy (HR-TEM) images of a region of the sample at two magnifications. (**d**) Energy dispersive X-ray spectroscopy (EDS) line-scan over three crystallites showing the homogeneous distribution of the two metals.

The powder XRD pattern of chromium substituted gallium oxide shows a highly broadened diffraction profile that can be indexed and fitted to cubic space group *Fd3m*, as shown in Figure 1a. The refined lattice parameter *a* = 8.233(2) Å is very similar to nanocrystalline γ-Ga_2_O_3_, which has a reported value of *a* = 8.2440(2) Å [13]. This is unsurprising given that the Cr^3+^ cation is very similar in size to the Ga^3+^ cation in an octahedral environment (0.615 Å for Cr^3+^ and 0.62 Å for Ga^3+^) [22]. The extremely broad Bragg reflections suggest a poorly crystalline material consisting of very small particles, and high-resolution transmission electron microscopy (HR-TEM) imaging confirms the small particle size with individual crystallites having a size around 5 nm in diameter, as shown in Figure 1b,c. Atomic resolution images show that the material has ordered crystalline lattice fringes, as shown in Figure 1b. EDS measured on the TEM confirmed the ratio of chromium to gallium in the spinel as approximately 3:7. As noted in the experimental section, this material represents the composition limit: no higher chromium content was achieved by this synthesis method. The homogeneity of distribution of Cr and Ga was proved by EDS line-scans, as shown in Figure 1d.

**Figure 2:**
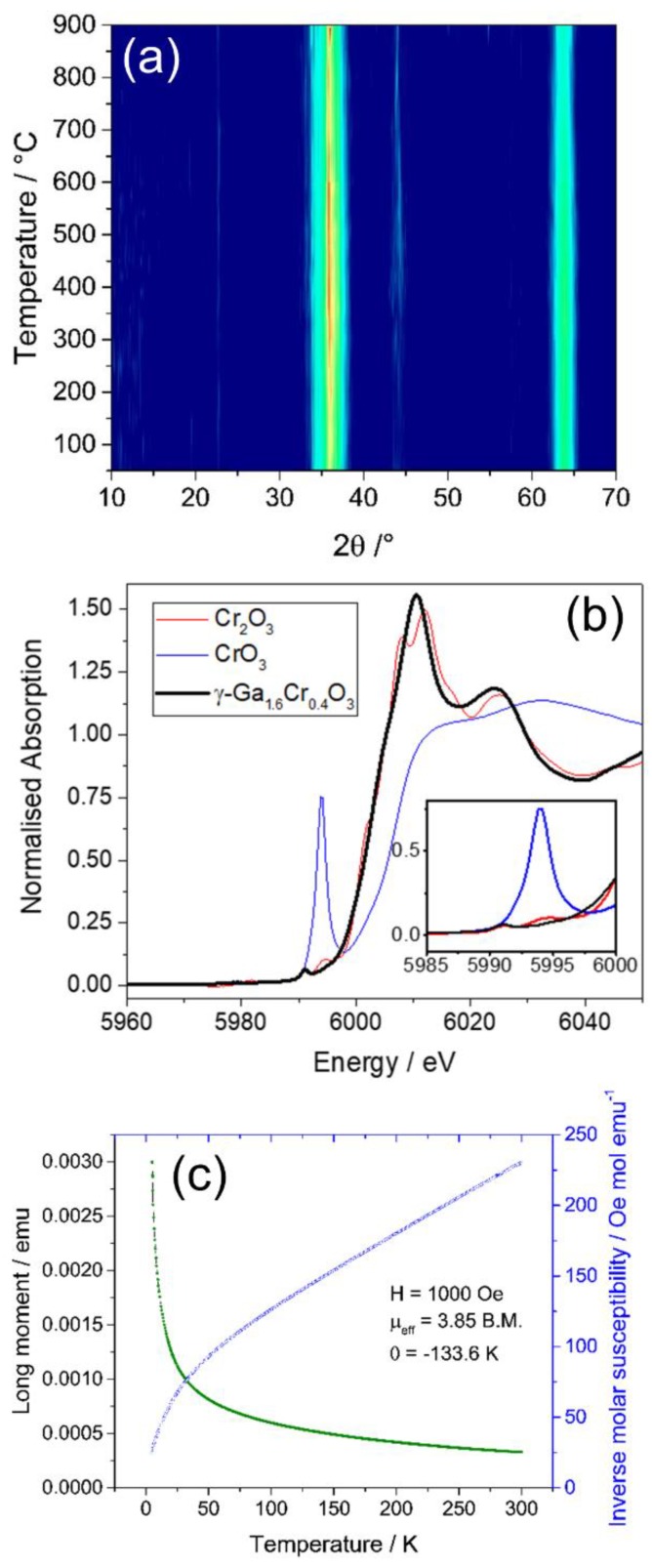
(**a**) In situ thermodiffractometry of γ-Ga_1.4_Cr_0.6_O_3_ showing lack of structure collapse to 900 °C; (**b**) Cr K-edge X-ray absorption near-edge spectroscopy (XANES) spectra of γ-Ga_1.4_Cr_0.6_O_3_ and two crystalline standards with (inset) showing the lack of a pre-edge feature in the spinel; (**c**) field cooled (FC) magnetometry of γ-Ga_1.4_Cr_0.6_O_3_ (green) showing simple paramagnetic behaviour, and inverse susceptibility (blue).

X-ray diffraction was measured as a sample of Ga_1.4_Cr_0.6_O_3_ that was heated to 900 °C at 50 °C temperature intervals, as shown in Figure 2a. The spinel structure was stable up to ~850 °C before phase separation began to occur, indicated by the appearance of reflections at 50° and 55° 2θ, which are most likely due to the emergence of hexagonal Cr_2_O_3_. Although some phase separation had occurred, the spinel structure was still present at 900 °C, and no β-Ga_2_O_3_—the thermodynamically stable polymorph of Ga_2_O_3_—was observed. This suggests that the Cr_2_O_3_ was ejected from the spinel structure at 850 °C and its presence as a secondary phase hindered the further transformation of the remaining gallium-rich phase. The substitution of chromium for gallium enhanced the thermal stability of the spinel as pure γ-Ga_2_O_3_ transformed into β-Ga_2_O_3_ above 700 °C [11]. This provides further evidence for the presence of a homogeneous single phase, and also shows how the presence of Cr stabilises the collapse to a β-Ga_2_O_3_ phase, and/or complete phase separation to hexagonal α-Cr_2_O_3_. 

Cr K-edge XANES spectroscopy, as illustrated in Figure 2b, shows that the chromium is present exclusively in the trivalent oxidation state. Examination of the pre-edge region shows the lack of any significant features, which is indicative of chromium substituting gallium for the octahedral sites in the spinel. Furthermore, the near edge structure is distinctly different than that of hexagonal α-Cr_2_O_3_, showing that the chromium was not phase-separated in the mixed oxide. The material can be described as a defect spinel, with the formula γ-Ga_1.4_Cr_0.6_O_3_ or 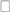
_0.33_Ga_1.867_Cr_0.8_O_4_, where the vacant sites, 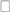, may be octahedral or tetrahedral, or a proportion of each. In our previous work we found that the parent γ-Ga_2_O_3_ spinel contained Ga^3+^ in octahedral and tetrahedral sites, with a distribution that depended on the synthesis method and particle size, but with predominantly octahedral for the most crystalline sample [13]. Since XANES evidence points to the presence of octahedral Cr^3+^, which is chemically the most likely coordination environment for this cation, we can assume that the mixed Cr-Ga spinel contains a higher proportion of tetrahedral gallium than the binary parent phase. 

The field cooled (FC) *M vs T* plot of γ-Ga_1.4_Cr_0.6_O_3_ shows that this material is paramagnetic, displaying linear Curie-Weiss behaviour to 50 K, as shown in Figure 2c. The effective magnetic moment of the Cr^3+^ cation in this material was calculated as 3.85 μ_B_, which is in good agreement with the spin only formula calculation for an octahedral d^3^ cation of 3.88 μ_B_. The negative Weiss constant suggests a tendency for the Cr^3+^ spins to align antiferromagnetically. To our knowledge, this is the first report of a ternary Cr-substituted gallium oxide material: other spinels that contain Cr and Ga are part of more complex solid solutions such as Ga*_x_*CoFe_1−*x*_CrO_4_ [23].

### 3.2. Mn-Ga_2_O_3_

**Figure 3:**
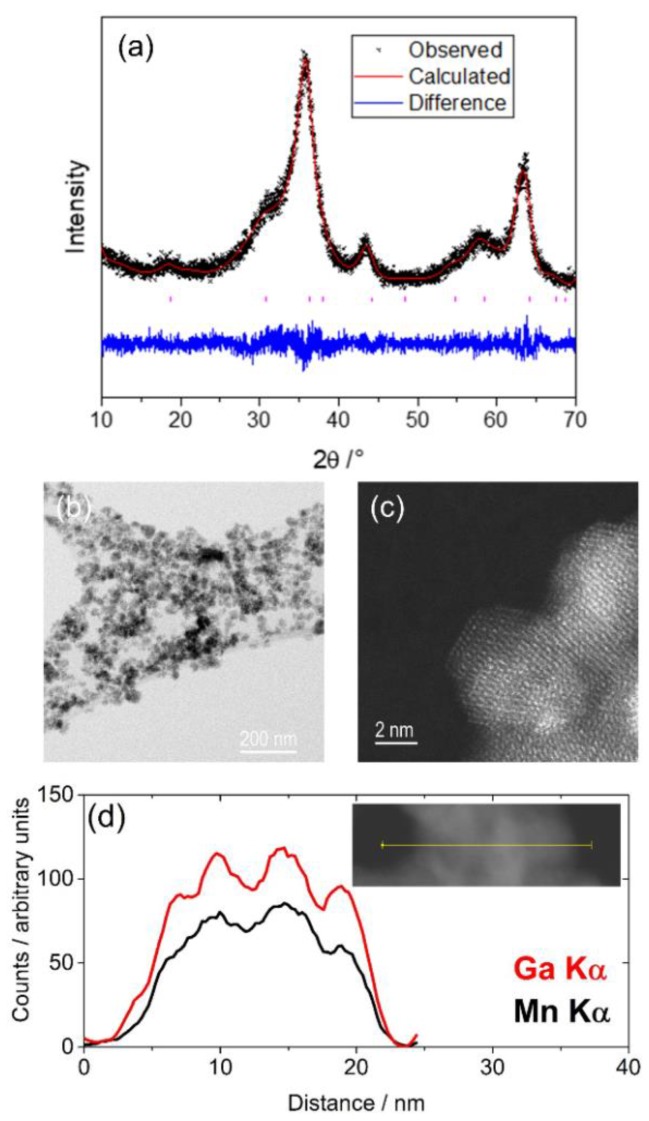
(**a**) Pawley refinement against powder X-ray diffraction of manganese gallium oxide prepared solvothermally; (**b**) and (**c**) TEM images of the material at two different magnifications, and (**d**) EDS line-scans showing the homogeneous distributions of the two metals.

The room temperature powder XRD pattern of the manganese gallium oxide material prepared is shown in Figure 3a. Very broad Bragg reflections suggest nano-sized particles, which was confirmed by TEM imaging, as shown in Figure 3b,c. The material prepared was apparently less crystalline than the pure nano-crystalline γ-Ga_2_O_3_ (prepared by a similar solvothermal reaction). The powder pattern can be indexed, and the profile fitted to a cubic spinel group with space group *Fd3m*, and a Pawley refinement of the lattice parameter showed an increase in the unit cell size compared to pure γ-Ga_2_O_3_ prepared by a similar solvothermal reaction, (8.297(4) *cf.* 8.258 Å, respectively). This is consistent with the larger size of the Mn^2+^ cation than the Ga^3+^ (Mn^2+^: 0.66 Å (tetrahedral), 0.83 Å (octahedral) *cf*. Ga^3+^: 0.47 Å (tetrahedral), 0.62 Å (octahedral) [22]), but is a smaller lattice parameter than reported for MnGa_2_O_4_ prepared by a high temperature synthesis (8.435 Å) [24]. A difference in the cation distribution in this poorly crystalline material prepared solvothermally compared to the arrangement prepared from high temperature synthesis could account for this observation (see below for further discussion).

**Figure 4:**
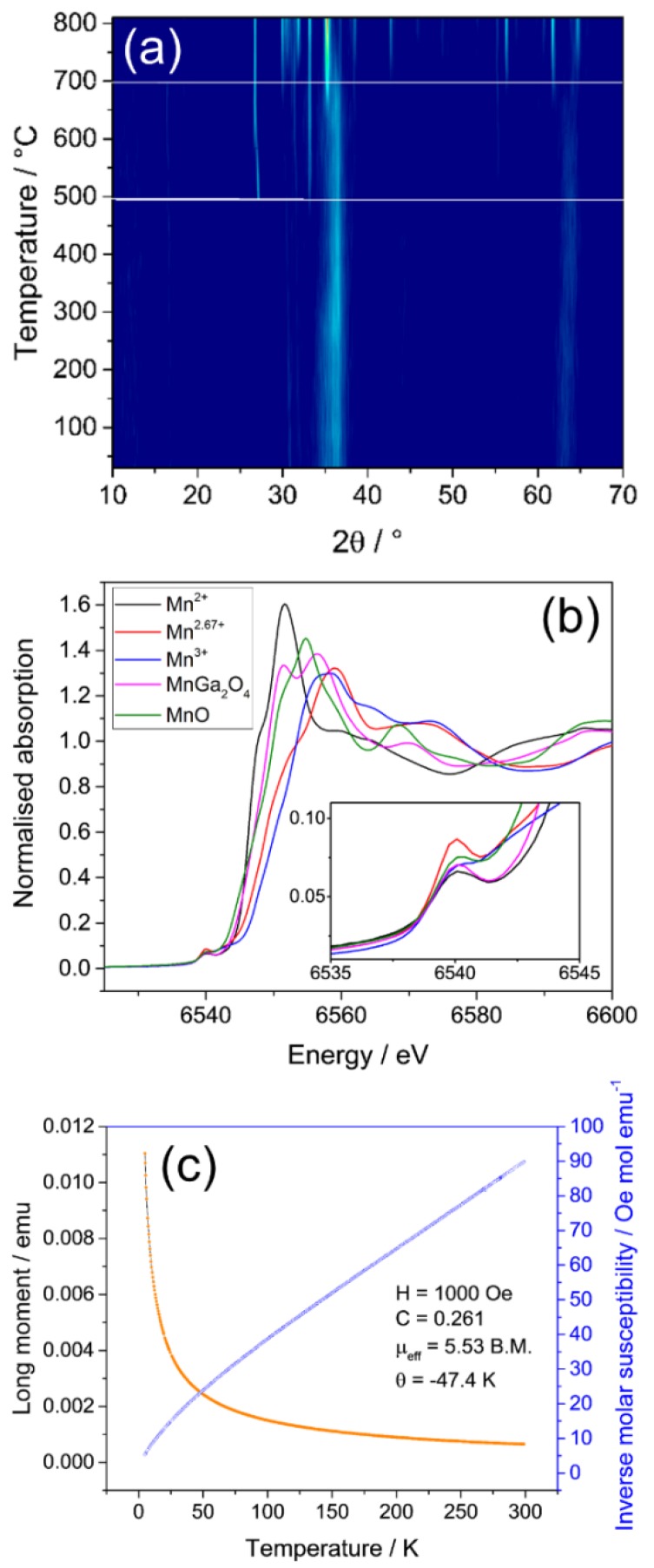
(**a**) In situ thermodiffractometry of manganese gallium oxide showing phase separation at 500 °C to Mn_2_O_3_ and then formation of β-Ga_2_O_3_ around 750 °C, (**b**) Mn K-edge XANES spectra of manganese gallium oxide with relevant reference samples, (Mn^2+^-Mn(NO_3_)_2_·4H_2_O; Mn^2.67+^-Mn_3_O_4_; Mn^3+^-Mn(acac)_3_) and (**c**) field cooled magnetometry of a sample of manganese gallium oxide (orange) displaying simple paramagnetic behaviour and inverse molar susceptibility (blue).

In situ X-ray thermodiffractometry shows that the poorly crystalline MnGa_2_O_4_ spinel begins to phase separate at ~480 °C, to first give Mn_2_O_3_, and then underwent phase transformation into β-Ga_2_O_3_ upon reaching 700 °C, as shown in Figure 4a. This is in contrast to the Cr-substituted material described above, and the redox chemistry of manganese may drive the phase separation of the mixed-oxide, unlike Cr^3+^, which is comparatively stable. XANES was measured at the Mn K-edge and suggests that in the manganese gallium spinel, the majority of manganese is present as Mn^2+^, with octahedral coordination as in rock salt MnO, as shown in Figure 4b. There is only a small pre-edge feature in the XANES, though this is not as intense as the pre-edge in Mn_3_O_4_ and so it is unlikely that there is much tetrahedrally coordinated manganese, indicating that the material prepared by this method was largely an inverse spinel, with Ga^3+^ distributed over both tetrahedral and octahedral sites and Mn^2+^ largely octahedral. This is in contrast to the MnGa_2_O_4_ reported the literature from high temperature synthesis, which contains ~70% tetrahedral Mn^2+^ and is therefore considerably less inverted [25]. This indicates that there is a different cationic distribution in the spinel prepared by solvothermal synthesis than that prepared by a solid-state reaction, and this would then explain the difference in the lattice parameter noted above.

Field cooled *M vs T* magnetometry of a sample of MnGa_2_O_4_ shows simple paramagnetic behaviour and displays linear Curie–Weiss behaviour between the temperature range 50–300 K, as shown in Figure 4c. The effective magnetic moment calculated was 5.53 μ_B_, which is slightly lower than the spin only value of 5.92 μ_B_ for a high spin d^5^ cation in octahedral coordination. This coordination is in agreement with the XANES analysis. A small negative Weiss constant is suggestive that the spins align antiferromagnetically.

MnGa_2_O_4_ has previously been prepared by high temperature solid state reactions and, as noted above, shown to be a largely normal spinel with Mn^2+^ occupying the tetrahedral sites [25,26]. The material showed antiferromagnetic ordering with a Néel temperature of 33 K [26]. One-dimensional nanostructures of MnGa_2_O_4_ and Zn-doped MnGa_2_O_4_ have been reported [27] by thermal evaporation techniques but the only solvothermal synthesis of MnGa_2_O_4_, to date, used microwave heating in the synthesis [28]. For our material, the presence of Mn^3+^ cannot be unequivocally ruled out from the XANES and also the slightly lower than expected magnetic moment per Mn may suggest some manganese in a higher oxidation state. We therefore used the method of Poix [29] to predict the lattice parameters of possible scenarios (see Supporting Information). For the case of the presence of solely Mn^2+^, the inverse spinel formulation Ga(III)_A_[Mn(II)Ga(III)]_B_O_4_ where A and B indicate tetrahedral and octahedral sites, respectively, the predicted lattice parameter of 8.44 Å is rather larger than the observed 8.297(4) Å (see above). On the other hand, if a proportion of octahedral Mn^3+^ is introduced, along with charge-balancing vacancies, then a small lattice parameter is predicted: for example, for the composition Ga(III)_A_[Mn(II)_0.47_Mn(III)_0.47_Ga(III)_0.88_
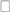
_0.18_]_B_O_4_ has a predicted lattice parameter of 8.36 Å. Additional evidence for the presence of Mn^2+^ in the new material was provided by thermogravimetric analysis in air, which showed mass gains at around 350 and 500 °C (see Supporting Information), consistent with the work of Laarj et al. on other manganese containing spinels [30], therefore, we conclude that the spinel from solvothermal synthesis contains a mixture of Mn^2+^ and Mn^3+^.

### 3.3. Fe-Ga_2_O_3_

**Figure 5:**
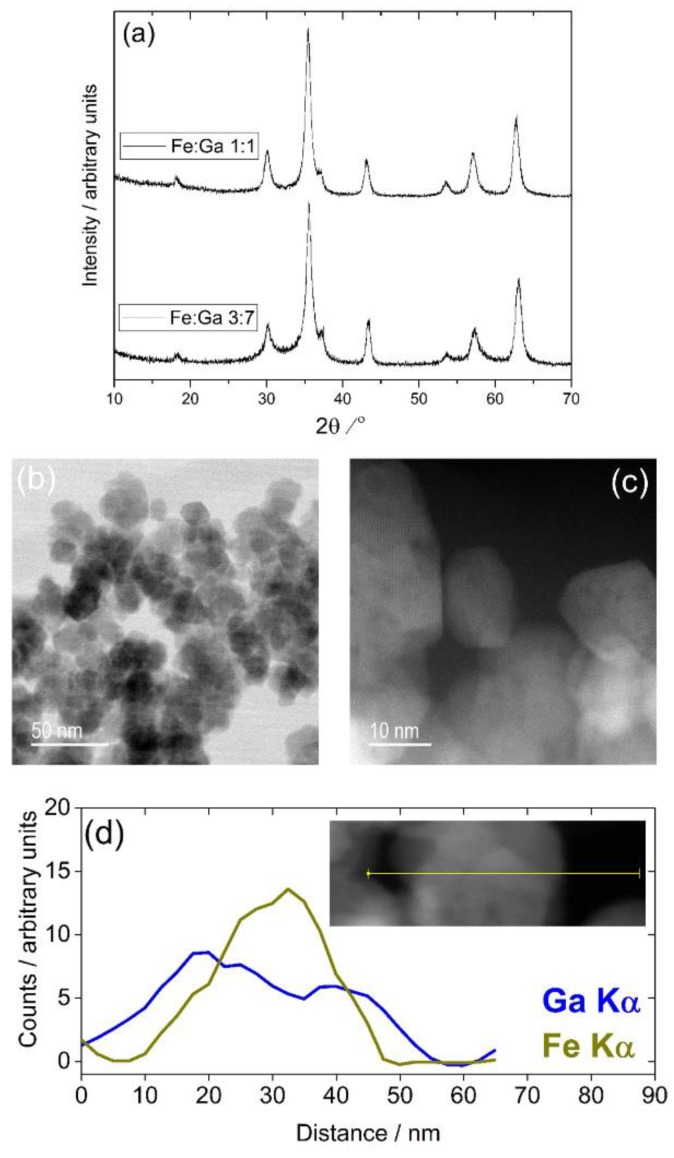
(**a**) Powder XRD of two samples of Fe-substituted Ga_2_O_3_ with different Fe:Ga ratios; (**b**,**c**) HR-TEM of the 3:7 Fe:Ga ratio material and (**d**) EDS line-scan of a region of the 3:7 Fe:Ga sample showing local inhomogeneity.

The iron-containing materials are somewhat more crystalline than the Cr- and Mn-substituted materials discussed so far, as shown by powder XRD in Figure 5a, while electron microscopy, shown in Figure 5b, confirms the presence of larger crystallites, around 20 nm in diameter, alongside smaller crystals of just a few nanometres in size. Scherrer analysis of the diffraction profile gives an average crystal domain size of 11.5 ± 1.5 nm, but this value should be treated with caution given the dispersity in crystallite size and the fact that elemental analysis using line-scans on the TEM reveals inhomogeneity in elemental distribution in regions of the sample with 7:3 Ga:Fe ratio, as shown in Figure 5d. This suggests the presence of Fe-rich regions of sample and Ga-rich regions. Interestingly, we previously found the same inhomogeneity in Fe-Ga oxides prepared from gallium metal in solvothermal reactions in ethanolamine/water mixtures [15], suggesting that the chemistry of their formation may be difficult control from solution.

**Figure 6:**
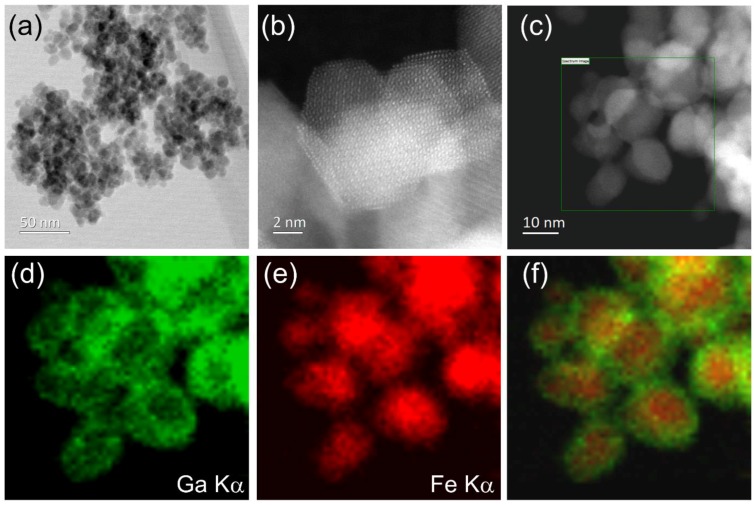
TEM of spinel phase with 1:1 Fe:Ga ratio. (**a**) Shows low magnification view, (**b**) higher magnification image, (**c**) region of elemental analysis, (**d**) EDS map for Ga, (**e**) EDS map for Fe and (**f**) composite elemental map. The box in (**c**) represents the regions studied in (**d**–**f**).

The sample prepared with a 1:1 ratio of Fe:Ga shows even more dramatic evidence of phase separation, with core-shell structures evident in EDS maps, as shown in Figure 6. This reveals gallium-rich shells on iron-rich cores even within the smallest particles of 2–3 nm in dimension.

**Figure 7:**
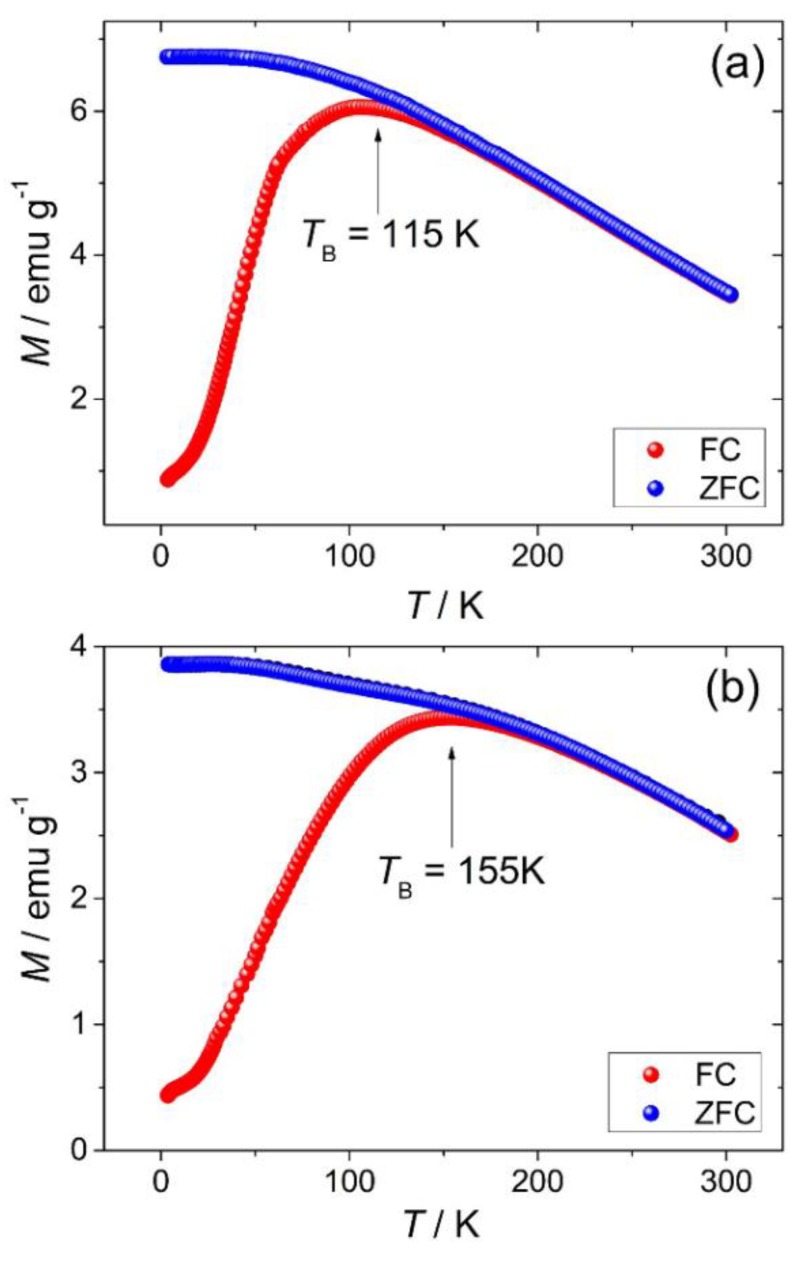
Magnetisation curves measured in zero-field cooling (ZFC, blue) and field cooling (FC, red) mode with *H*_FC_ = 100 Oe for Fe substituted γ-Ga_2_O_3_ (**a**) Fe:Ga 1:1 and (**b**) Fe:Ga 3:7. *T*_B_ indicates the blocking temperatures.

**Figure 8:**
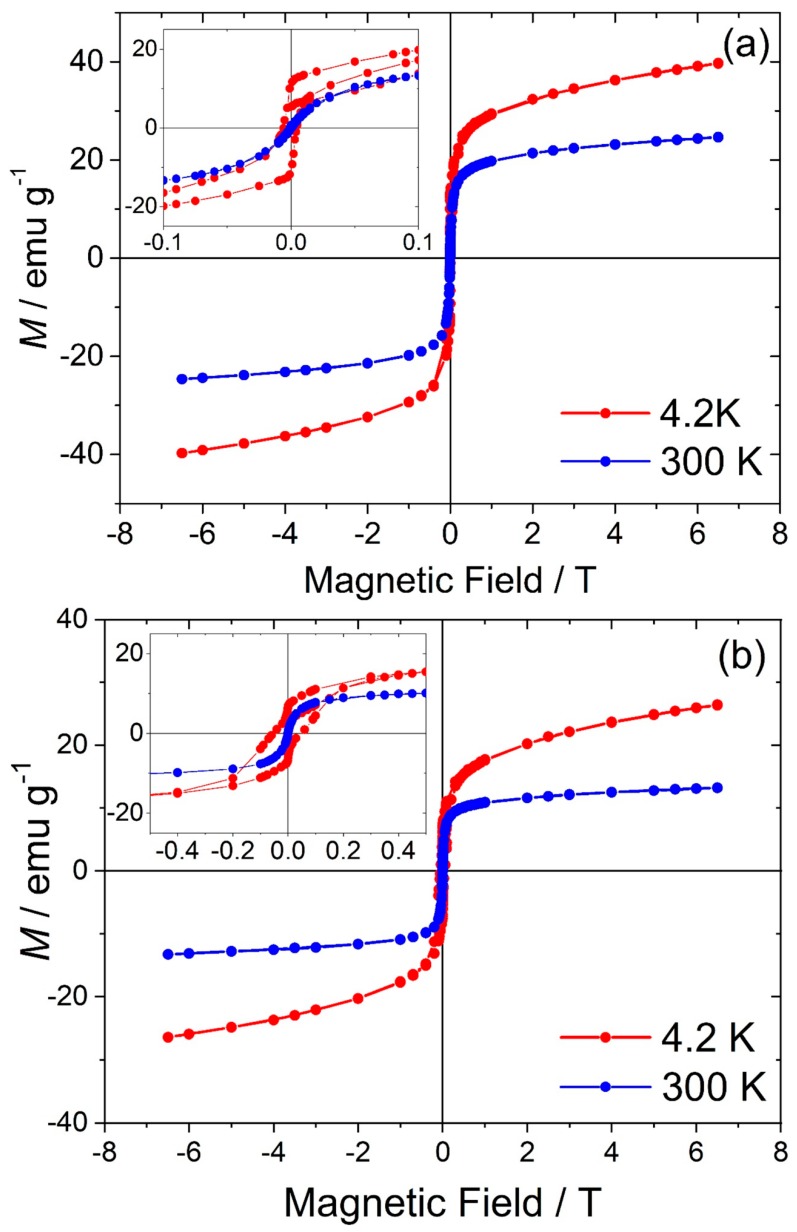
Hysteresis curves measured at 4.2 K (red) and 300 K (blue) of Fe substituted γ-Ga_2_O_3_ for samples (**a**) Fe:Ga 1:1 and (**b**) Fe:Ga 3:7. The insets show expansion of the curves in the low-field region.

Magnetisation measurements *M vs T* of Fe substituted γ-Ga_2_O_3_ samples are shown in Figure 7. For the zero-field cooling, the samples were first measured in zero-field from room temperature down to 4.2 K (ZFC), after a field of 100 Oe was applied, and sample temperature increased to 300 K. After this process, samples were cooled and measured again down to 4.2 K under the applied field (FC). Comparing the magnetisation curves of both samples Fe:Ga with a ratio 1:1 as shown in Figure 8a, and 3:7 as shown in Figure 8b, we note superparamagnetic behaviour for both samples in the upper temperature range revealing blocking temperatures of about 115 and 155 K, respectively. 

Figure 8 shows hysteresis loops of both Fe-substituted γ-Ga_2_O_3_ samples with expanded regions for low-fields shown in the insets of the Figure 8a,b for samples with Fe:Ga ratios of 1:1 and 3:7, respectively. While at room temperature no hysteresis is observed, showing that both samples were completely in a superparamagnetic regime, at 4.2 K coercivity fields *H*_C_ are 100 Oe and 300 Oe, respectively, indicating ferromagnetic-like behaviour at low temperatures. On the other hand, the *M vs T* curves also show that even at the highest field available (7 T) the magnetisation was still not saturated indicating other contribution from paramagnetic phases [31,32].

**Figure 9:**
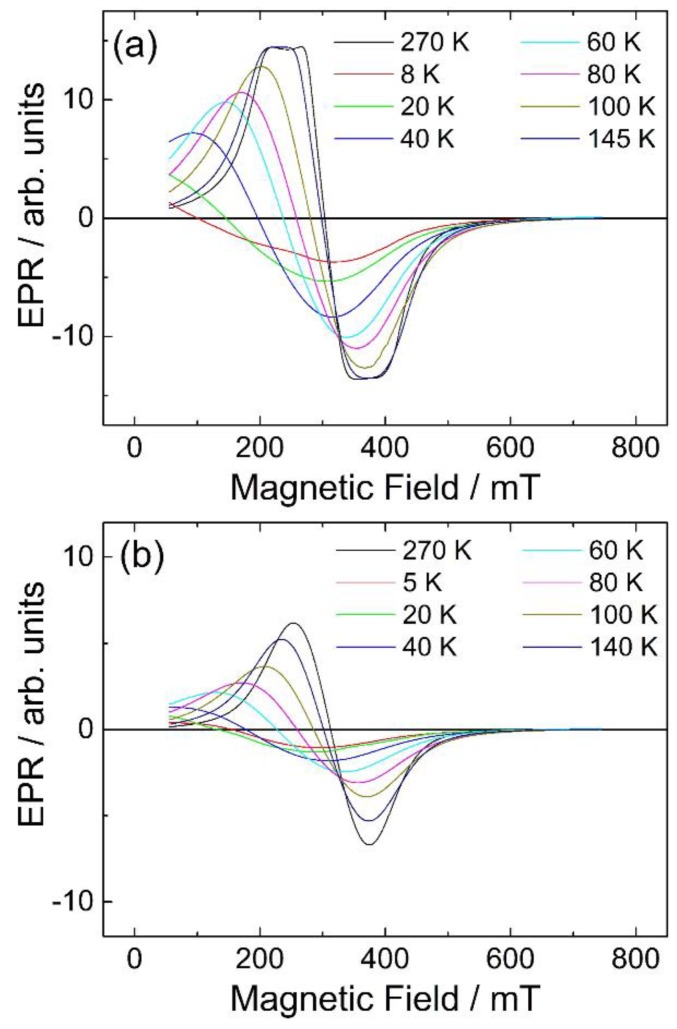
Electron paramagnetic resonance (EPR) spectra measured at 9.44 GHz as a function of temperatures for Fe substituted γ-Ga_2_O_3_ (**a**) Fe:Ga 1:1 and (**b**) Fe:Ga 3:7.

Electron paramagnetic resonance measurements of both samples as a function of temperature are shown in Figure 9. The spectra present asymmetric derivative line shapes and the centre positions shift to lower magnetic fields with decreasing temperatures, typical for superparamagnetic particles, which enter into a ferrimagnetic regime at low temperatures.

**Figure 10:**
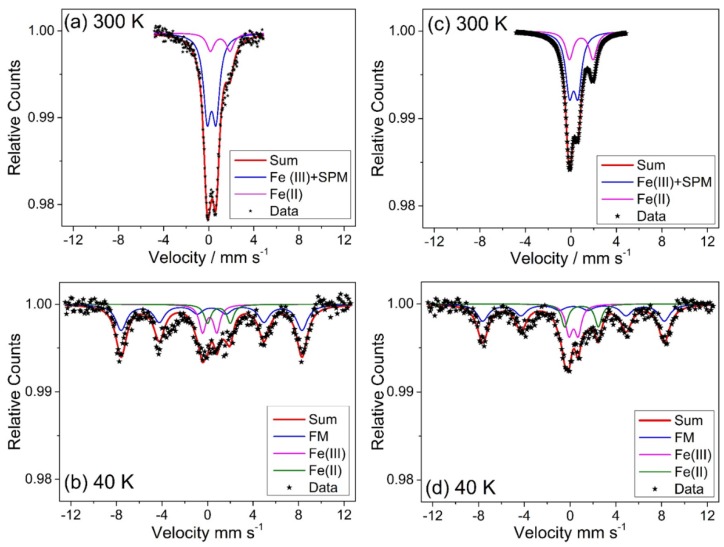
^57^Fe Mössbauer spectra of the Fe substituted γ-Ga_2_O_3_ measured at 300 K and 40 K for samples with an Fe:Ga ratio of 1:1 (**a**,**b**) and a ratio of 3:7 (**c**,**d**), respectively. SPM indicates superparamagnetic contribution at 300 K.

In order to investigate in more detail local symmetry, valence states and magnetic ordering of Fe ions in the two Fe substituted γ-Ga_2_O_3_ samples with an Fe:Ga ratio of 1:1 and 3:7, ^57^Fe Mössbauer spectra were measured at different temperatures. This spectroscopic technique is sensitive to all types of iron present in the samples and it is appropriate to determine the relative concentrations of different phases and valence states of iron. Figure 10 shows the ^57^Fe Mössbauer spectra for both samples obtained at room temperature and at 40 K. 

While the room temperature spectra are described by the superposition of two or more doublets, the spectra measured at 40 K were dominated by a sextet hyperfine-split spectrum. This observation is typical for superparamagnetic particles (room temperature) that are blocked at low temperature representing a ferromagnetic state. The parameters of the fit are shown in Table 1. It should be noted that the individual linewidth of the fits were large (~0.8 mm s^−1^), which may indicate distribution of different particle sizes local distribution of Fe inside the particles. This is frequently observed in mixed iron-containing spinel nanoparticles [33].

The important point of this analysis is that the doublet structure observed at room temperature in both samples with isomer shift δ of about 0.25(1) mm s^−1^ and quadrupole shift of about 0.77(2) mm s^−1^ was dominantly due to superparamagnetic particles, which was transformed into a sextet structure at 40 K with isomer shift δ of about 0.37(2) mm s^−1^, zero quadrupole shift and hyperfine interaction of 49.3(1) T [34]. The sextet parameters are typical for magnetite (Fe_3_O_4_) nanoparticles below the Verwey transition (~122 K) for which the individual tetrahedral and octahedral Fe sites cannot be distinguished [35]. 

Ideal magnetite has the cubic inverse spinel structure. For the inverse spinel the divalent ions are exchanged with trivalent ions from the tetrahedral to octahedral site. The formula of magnetite is commonly written as Fe(III)_A_[Fe(II),Fe(III)]_B_O_4_ where A and B indicate tetrahedral and octahedral sites, respectively. For our samples of iron substituted γ-Ga_2_O_3_, it was most probably that Ga(III), to some parts was exchanged by Fe(III). This mixed magnetite may be written as [Fe(III),Ga(III)]_A_[Fe(II),Fe(III),Ga(III)]_B_O_4_. This mixed magnetite phase represents about 73% and 67% in the samples with Fe:Ga ratios of 1:1 and 3:7, respectively. 

The ^57^Fe Mössbauer spectra and their fits (see parameters in Table 1) show that apart from the superparamagnetic doublet (300 K), which was transformed at low temperature (40 K) into the sextet structure, two doublets remain that represent about 1/3 of the total iron. One of the doublets with isomer shift δ of 0.95(6) mm s^−1^ and quadrupole shift ε of about 1.85(15) is typical for Fe(II) in octahedral symmetry. The other doublet was strongly superimposed on the superparamagnetic spectrum at room temperature and somewhat better resolved at 40 K, with Mössbauer parameters similar to that of the superparamagnetic phase and is typical for Fe(III) in octahedral symmetry. Both these doublets represent a paramagnetic phase and for our samples may be represented in an Fe diluted mixed Ga-rich phase. The ^57^Fe Mössbauer spectroscopy hence confirms the TEM findings of phase separation on the nanoscale, with regions of Fe-rich and Ga-rich spinel oxides. 

## 4. Conclusions

The use of acetylacetonate precursors and organic solvents allows a convenient route to mixed-metal spinels under solvothermal conditions. This has allowed the first ternary Cr-Ga oxide to be isolated and new variants of Mn- and Fe-substituted spinels to be crystallised that show different distributions of cations compared to previously reported materials from high-temperature solid-state synthesis. The materials are formed directly from solution as nanoscale crystallites, which may be desirable for future applications, where their high surface:bulk structure may be useful for catalysis, or dispersion in a medium may allow convenient processing for device fabrication. The observation of nanoscale phase separation and the formation of core-shell like structures for mixed Fe-Ga oxides is likely to arise from the solution synthesis method but is a potentially useful method for control of chemical and physical properties, and in future work we will investigate whether this can be controlled in a rational way by tuning the synthetic chemistry.

## Figures and Tables

**Table 1:** ^57^Fe Mössbauer parameters: isomer shift (*δ*), quadrupole shift (*ε*), hyperfine interaction (*B*_HF_) and total area for different Fe-related phases in Fe substituted γ-Ga_2_O_3_ materials with Fe:Ga 1:1 and Fe:Ga 3:7.

Sample	*T*/K	*δ*/mm s^−1^ (± 0.08)	ε/mm s^−1^ (± 0.15)	*B*_HF_/T (± 0.05)	Area (%) (± 1)
(Fe:Ga) (1:1)	300	0.25	0.79	-	80
		1.04	1.77	-	20
	40	0.35	0.00	49.4	73
		0.35	0.80	-	15
		0.80	2.30	-	12
(Fe:Ga) (3:7)	300	0.24	0.75	-	67
		0.91	2.07	-	33
	40	0.39	0.00	49.2	66
		0.30	0.78	-	25
		0.72	2.30	-	9
